# The Cerebellar Predictions for Social Interactions: Theory of Mind Abilities in Patients With Degenerative Cerebellar Atrophy

**DOI:** 10.3389/fncel.2018.00510

**Published:** 2019-01-08

**Authors:** Silvia Clausi, Giusy Olivito, Michela Lupo, Libera Siciliano, Marco Bozzali, Maria Leggio

**Affiliations:** ^1^Ataxia Laboratory, IRCCS Fondazione Santa Lucia, Rome, Italy; ^2^Department of Psychology, Sapienza University of Rome, Rome, Italy; ^3^Neuroimage Laboratory, IRCCS Fondazione Santa Lucia, Rome, Italy; ^4^PhD Program in Behavioral Neuroscience, Sapienza University of Rome, Rome, Italy

**Keywords:** cerebellum, cerebro-cerebellar networks, VBM, SBA, theory of mind, prediction, social interaction

## Abstract

Recent studies have focused on the role of the cerebellum in the social domain, including in Theory of Mind (ToM). ToM, or the “mentalizing” process, is the ability to attribute mental states, such as emotion, intentions and beliefs, to others to explain and predict their behavior. It is a fundamental aspect of social cognition and crucial for social interactions, together with more automatic mechanisms, such as emotion contagion. Social cognition requires complex interactions between limbic, associative areas and subcortical structures, including the cerebellum. It has been hypothesized that the typical cerebellar role in adaptive control and predictive coding could also be extended to social behavior. The present study aimed to investigate the social cognition abilities of patients with degenerative cerebellar atrophy to understand whether the cerebellum acts in specific ToM components playing a role as predictive structure. To this aim, an *ad hoc* social cognition battery was administered to 27 patients with degenerative cerebellar pathology and 27 healthy controls. In addition, 3D T1-weighted and resting-state fMRI scans were collected to characterize the structural and functional changes in cerebello-cortical loops. The results evidenced that the patients were impaired in lower-level processes of immediate perception as well as in the more complex conceptual level of mentalization. Furthermore, they presented a pattern of GM reduction in cerebellar portions that are involved in the social domain such as crus I-II, lobule IX and lobule VIIIa. These areas showed decreased functional connectivity with projection cerebral areas involved in specific aspects of social cognition. These findings boost the idea that the cerebellar modulatory function on the cortical projection areas subtends the social cognition process at different levels. Particularly, regarding the lower-level processes, the cerebellum may act by implicitly matching the external information (i.e., expression of the eyes) with the respective internal representation to guarantee an immediate judgment about the mental state of others. Otherwise, at a more complex conceptual level, the cerebellum seems to be involved in the construction of internal models of mental processes during social interactions in which the prediction of sequential events plays a role, allowing us to anticipate the other person's behavior.

## Introduction

Estimation of mental states of others is a key aspect for human communication and social interactions. This capacity is a fundamental component of the social cognition and involves both lower-level processes of immediate perception and higher-level inferences (Coricelli, 2005; Van Overwalle et al., 2014). The lower-level processes are automatic, refer to a primitive understanding of another person's mind and are based on action and emotion recognition and “emotional contagion” (Meltzoff and Moore, 1989). The higher-level inferences imply the capacity to attribute mental states to others (such as emotion, intentions and beliefs) and adopting the perspective of the other person to understand and predict the behavior (Van Overwalle et al., 2014). This ability is known as Theory of Mind (ToM) (Premack and Woodruff, 1978; Brothers and Ring, 1990) or the “mentalizing” process and is based on intentionality, empathy, and higher depths of reasoning, requiring more conceptual and voluntary processes (Coricelli, 2005).

Such complex functions require a correspondent sophisticated neural mechanism. Indeed, complex interactions between limbic, associative areas and subcortical structures are crucial to these processes (Van Overwalle et al., 2014; Van Overwalle and Mariën, 2016; Heleven and Van Overwalle, 2018). Within the social cognition domain, ToM abilities seem to mainly depend on a group of brain regions, called the “mentalizing network,” which includes regions in the superior temporal sulcus (STS), temporoparietal junction, medial precuneus, and medial prefrontal cortex (Saxe and Kanwisher, 2003; Aichhorn et al., 2009). The neural circuitry underlying social cognition also involves fronto-limbic connections (Beer et al., 2006), mirror neurons in the ventral premotor and rostral posterior parietal cortices (Rizzolatti et al., 2006), the amygdala (Adolphs, 2004), the insula (Kipps et al., 2007; Gu et al., 2012), and the middle temporal gyrus (Johnstone et al., 2006).

Most neuroanatomical models of social behavior/mentalizing emphasize the putative role of the cortical regions (Abu-Akel and Shamay-Tsoory, 2011) and fail to acknowledge the contribution of the cerebellum. However, recent studies have focused on the role of the cerebellum in the social domain, including some aspects of ToM (Sokolov, 2018).

The revolutionary view of a “social cerebellum” is supported by observations of cerebellar activation in many functional imaging studies involving social emotions and mental state inference tasks (Brunet et al., 2000; Calarge et al., 2003) as well as by findings showing that the performance of patients with cerebellar damage is impaired in a range of perceptual (Ivry and Keele, 1989; Ackermann et al., 1997), cognitive (Schmahmann and Sherman, 1998; Tedesco et al., 2011), and ToM tasks (Sokolov, 2018) that are essential in social interactions. In particular, alterations in social cognition tasks are reported in patients with complex cerebello-cerebral degeneration, such as spino-cerebellar ataxia (SCA) type 1, SCA type 2, and SCA type 7 as well as in patients with isolated cerebellar degeneration, such as SCA type 6, SCA type 8 and episodic ataxia type 2 (Sokolovsky et al., 2010; D'Agata et al., 2011; Hoche et al., 2016).

Moreover, cerebellar abnormalities and dysfunctions of cerebellar-cortical networks have been described in several psychiatric disorders characterized by mentalizing impairments (i.e., schizophrenia and autism spectrum disorders) (Andreasen and Pierson, 2008; Fatemi et al., 2012).

The cerebellar function in social behavior is anatomically supported by the fact that the cerebellum is incorporated into associative and paralimbic circuits involved in social cognition processes by way of feedforward connections from these cerebral cortical areas to the cerebellum via the pons (corticopontocerebellar projections) and by feedback connections from the cerebellum through the thalamus back to the cerebral cortex (cerebellothalamocerebral projections) (Schmahmann and Pandya, 1997; Ramnani, 2012).

Although there is widespread agreement about the neural substrate of social cognition, much less is known about the neural representations and computations that are implemented in cerebello-cerebral circuitries. In particular, the specific role of the cerebellum in the social domain remains to be elucidated.

As it is well acknowledged, cerebellar operations in the sensorimotor domain are believed to involve outcome prediction based on forward models and signaling deviations from these outcomes (prediction errors) to the cerebral cortex (Ito, 2006). In particular, the cerebellum receives and combines the motor commands with exteroceptive and proprioceptive sensory inputs, generating a representation of the expected sensory consequences of those commands (internal models) (Miall and Reckess, 2002). Therefore, the sensory predictions generated by a forward model can be used to coordinate motor output, providing a means to anticipate the consequences of a motor command and to update a state estimate of the motor system. These predictions are constantly compared with afferent input, and in the presence of deviations from prediction, the cerebellum emits corrective signals. These error signals allow us to refine future sensory predictions and reduce the prediction error signal on subsequent movements (Wolpert and Kawato, 1998).

In the present work, we followed the hypothesis that the typical cerebellar role in adaptive control and predictive coding in the sensorimotor domain could be extended to the social cognition domain (Ito, 2008; Sokolov, 2018). Indeed, anticipation, adaptation and learning appear indispensable for successful social interactions. Particularly, prediction is a central component of socioemotional processing (Brown and Brüne, 2012; Koster-Hale and Saxe, 2013) in the sense that the understanding and inference of another individual's state of mind requires not only the creation of a mental model of that mental state but also the ability to simulate how it might influence the others' behavior. Recognizing deviations from our expectation in the outcome of a social interaction and using that information to calibrate future social predictions guarantee adaptive social behavior (Sokolov et al., 2017).

In the complex mentalizing process, the predictions are allowed by stored internal models of human behaviors based on expectations that actions will be rational and efficient and consistent with individual beliefs, personality traits, or social norms (Koster-Hale and Saxe, 2013). Thus, in analogy with the information processing in the sensorimotor domain, the cerebellum might modulate the high-order cortical activity (Middleton and Strick, 2000) by detecting predictable sequences (i.e., internal model of a social action) and allowing optimized feedforward control that is necessary to accomplish these functions in a fluid and automated manner (Leggio et al., 2011; Leggio and Molinari, 2015). If this is the case, a cerebellar malfunction that interferes with using the internal model would prevent the prediction function and the correct inferences about the others' mental state or the recognition of a deviance from the expected social behavior.

In the present work, we investigated the social cognition abilities of patients with degenerative cerebellar atrophy to understand whether the cerebellum plays a role in particular components of social cognition and to elucidate its role as a predictor in social interactions. To this aim, the participants were tested using an *ad hoc* social cognition battery to examine the unconscious and automatic process and the more complex and conscious aspects of ToM by using tasks in which the stimuli implied different levels of prediction. Considering the etiological heterogeneity of the cerebellar disease in the present population, a morpho-volumetric analyses was also performed to characterize the common cerebellar structural changes and their neuroanatomical localization. Moreover, considering that meta-analytic connectivity data in healthy subjects and studies in patients affected by SCA2 indicated interactions between the cerebellum and cerebral areas that are crucial in social cognition (Habas et al., 2009; Van Overwalle et al., 2015; Olivito et al., 2017), functional connectivity (FC) between the common cerebellar areas affected in our sample and the cerebral cortex was analyzed by means of resting-state functional magnetic resonance imaging (RS-fMRI) (Friston et al., 1993; Biswal et al., 1997; van de Ven et al., 2004).

We expected that the cerebellar structural alterations that occurred in patients affected by cerebellar degeneration would interfere with the modulatory function of the cerebellum on the cortical projection areas involved in the mentalizing process. This interference could account for specific impaired ToM outcomes, particularly when the stimuli processing requires a high level of prediction.

## Materials and Methods

### Participants

Twenty-seven patients affected by degenerative cerebellar atrophy (CB) [mean age/SD: 46.4/10.8 (years); mean education/SD: 13.1/3.3 (years); M/F: 6/21] were recruited at the Ataxia Lab of the Santa Lucia Foundation Hospital. They were selected from among those in-patients and out-patients admitted between the 2014 and 2017 (n. 38) for rehabilitation or clinical follow up. Only the patients presented with diffuse cerebellar atrophy and no other brain macroscopic abnormalities, as detected by visual inspection of clinical MRI scans, were enrolled in the study.

At the time of the assessment, all the 27 patients had more than 6 months of illness from the diagnosis and showed a pure cerebellar motor syndrome, with no extra-cerebellar symptoms, as evidenced by a comprehensive neurological examination. The International Cooperative Ataxia Rating Scale (ICARS, Trouillas et al., 1997) was used to quantify the cerebellar motor signs. The demographic and clinical characteristics of the patients are reported in Table 1.

**Table 1:** Clinical and demographic characteristics of the cerebellar patients.

	**ID**	**Diagnosis**	**Gender**	**Age** ** (years)**	**Education** ** (years)**	**Duration** ** (months)**	ICARS^*^	**Triplet expansions**
1	CB1	FRDA	F	47	13	24	59	–
2	CB3	SCA2	F	38	12	12	33	CAG 22/41
3	CB4	SCA2	F	42	13	12	47	CAG 22/39
4	CB5	ICA	F	53	11	7	21	–
5	CB7	Cerebellitis	F	59	13	-	12	–
6	CB9	SCA2	F	44	18	13	26	CAG/CTG 14/47
7	CB12	ICA	F	59	13	8	16	–
8	CB13	ICA	F	56	13	17	29	–
9	CB14	ICA	F	52	13	44	28	–
10	CB15	SCA1	F	24	16	12	33	CAG 27/57
11	CB16	SCA2	F	36	13	8	37	CAG 22/42
12	CB17	ICA	F	24	13	10	8	–
13	CB18	ICA	F	46	13	24	9	–
14	CB20	SCA15	F	51	14	48	44	ITPR1 gene Heterozygous deletions
15	CB21	SCA2	F	54	18	12	27	CAG 22/37
16	CB22	SCA28	F	42	18	–	21	–
17	CB23	SCA15	F	56	13	–	35	ITPR1 gene Heterozygous deletions
18	CB24	SCA2	F	60	8	48	31	CAG 22/37
19	CB26	FRDA	M	29	13	48	25	–
20	CB27	SCA2	M	40	8	36	18	CAG 22/38
21	CB29	SCA2	M	64	17	36	27	CAG 22/35
22	CB30	SCA2	F	43	13	12	28	CAG
23	CB31	ICA	F	62	18	–	17	–
24	CB32	SCA1	M	45	8	48	33	CAG/CTG 18/58
25	CB33	SCA2	M	42	8	12	24	CAG 22/39
26	CB34	SCA2	M	42	18	12	17	CAG 22/39
27	CB35	ICA	F	44	8	–	33	–

The table reports for each patient diagnosis, gender, age, education, disease duration, the total motor scores as assessed by the International Cooperative Ataxia Rating Scale (ICARS) (Trouillas et al., 1997) and the CGA repeats.

* *ICARS: minimum score 0 (absence of motor deficits), maximum score 100 (maximum presence of motor deficits); FRDA, Friedreich's ataxia; SCA1, spinocerebellar ataxia type 1; SCA2, spinocerebellar ataxia type 2; SCA15, spinocerebellar ataxia type 15; SCA28, spinocerebellar ataxia type 28; ICA, Idiopathic Cerebellar Atrophy; F, Female; M, Male*.

Additionally, 27 well-matched healthy subjects (HS) [mean age/SD: 45.9/9.7 (years); mean education/SD: 13.1/2.6 (years); M/F: 6/21] with no history of neurological or psychiatric illness were enrolled in the study. *T*-test analyses showed no significant difference in the mean age (*t* = 0.17; *p* = 0.62) and educational level (*t* = −0.05; *p* = 0.20) between the two groups. Raven's 47 Progressive Matrices test (Raven, 1949) was administered to assess intellectual level and used as inclusion criterion.

The Ethics Committee of Fondazione Santa Lucia (IRCCS) approved the present study, according to the principles expressed in the Declaration of Helsinki, and written informed consent was obtained from all the participants.

### Neuropsychological Screening

A neuropsychological battery was administered to the CB patients to assess the following domains: current intellectual functioning [Wechsler Adult Intelligence Scale–Revised (Wechsler, 1981; Orsini and Laicardi, 1997)]; verbal comprehension [Token test (De Renzi and Vignolo, 1962)]; verbal production [Denomination of words subtest of the BADA–Batteria per l'Analisi dei Deficit Afasici–(Miceli et al., 1994); Phrase Construction subtest of the Italian-language Mental Deterioration Battery (Caltagirone et al., 1995)]; verbal memory [Immediate and Delayed recall of Rey's 15 words (Rey, 1958); forward and backward digit span (Wechsler, 1945; Orsini et al., 1987)]; episodic memory [Short-Story Recall task (Carlesimo et al., 2002)]; visuospatial memory [Rey-Osterrieth Complex Figure Test (recall) (Caffarra et al., 2002); Corsi Test Corsi, 1972]; visuospatial ability [Rey-Osterrieth Complex Figure (copy) (Caffarra et al., 2002)]; attention [Multiple features targets cancellation task (Marra et al., 2013); Lines cancellation task (Albert, 1973); Trail Making Test (Giovagnoli et al., 1996)]; and executive functions [Phonological fluency (Borbowsky et al., 1967); Wisconsin Card Sorting Test (Heaton, 1981)].

### Social Cognition Tasks

To investigate social cognition abilities, the following tests were administered.

The Reading the Mind in the Eyes test (RME) (Baron-Cohen et al., 2001; Serafin and Surian, 2004) was used to assess the automatic lower-level processes of emotion and mental state attribution based on immediate perceptions of the eye-region expression and regardless of the context. Indeed, within the face, the eyes are the most important contact between agents (Hainline, 1978; Maurer, 1985). This test was made up of 36 photos of actors' eyes, and for each, the participants had to choose from four alternative words the one that best described what the person in the photograph is thinking or feeling. This process is assumed to involve an unconscious, automatic and rapid matching of past memories/categorization concerning similar expressions with a lexicon of mental state terms to arrive at a judgment of which word the eyes most closely match (Baron-Cohen et al., 2001). Responses were scored 1 or 0 for correctness.

The Emotion Attribution test (EA) (Blair and Cipolotti, 2000; Prior et al., 2003) was used to assess the ability to attribute emotions to others in a social context. Fifty-eight short stories describing an emotional situation were presented to the subject and required providing a one-word description of how the main character might feel in that situation. The sentences were designed to elicit sadness, fear, embarrassment, disgust, happiness, anger or envy. The sequential events of the story were explicit and univocal, requiring a low level of prediction about the emotional consequences of the event (see Appendix in Supplementary Material for example of the story). The correct answer was based on the coherent expectation about the social interaction.

The Faux Pas test (FP) (Stone et al., 1998; Liverta Sempio et al., 2005) was used to assess a more advanced capacity to make inferences regarding another person's state of mind. This test included 10 stories in which a social “faux pas” occurred (“faux pas” stories) and 10 control stories in which no social “faux pas” occurred (“no-faux pas” stories) (see Appendix in Supplementary Material for examples of the stories).

A social “faux pas” occurs when a speaker says something without considering that the listener might not want to hear it or might be hurt by what has been said, implying false or mistaken belief. To recognize the “faux pas,” the subject had to understand that the person committing the faux pas does not know that they should not say it and that the person hearing it would be upset by the faux pas. Moreover, the subject had to identify a wrong behavior or action with respect to the predicted social norms or a more likely behavior in the social interaction. In the “faux pas” stories, the sequential events are unexpected and not univocal and a constant comparison between the event and the social expectation are necessary, thus requiring a high level of prediction. Conversely, in the “no-faux pas” stories, the sequential events are explicit and univocal, requiring a low level of prediction about the consequences of the event.

All the stories were read to participants, while they had a copy of the story to read along and check back over (to reduce the memory requirement). When a “faux pas” was identified, five clarifying questions were proposed to evaluate the understanding of the mental states and emotions of the agents involved in the stories. Each “faux pas” story question correctly answered was scored as 1, resulting in a maximum score of 6 for each story.

The “no-faux pas” stories were given a score of 2 if they were correctly identified as not containing a faux pas. Two more control questions were asked for all 20 stories to confirm that the participant had a factual understanding of the stories.

The Advanced ToM task (Happè, 1994; Blair and Cipolotti, 2000; Prior et al., 2003; Van Harskamp et al., 2005) was used to assess the more advanced concepts of ToM, such as double bluff, white lies, and persuasion. The participant was presented with 13 stories describing naturalistic social situations and was asked to interpret and justify the behavior of the main character. The subject had to accurately identify the underlying intention behind a character's utterance that was not literally true and to explain why the main character acted in a particular manner. Successful performance required the attribution of mental states, such as desires, beliefs or intentions, and higher-order mental states, such as one character's belief about what another character knows. The sequential events of the story were not univocal as in the FP stories, requiring a high level of prediction about the consequence of the events. The correct answer was based on the capacity to make a choice taking into account different expectations about the social interaction (see Appendix in Supplementary Material for examples of the stories).

### Visual Analog Scales for Mood and Anxiety

The possible anxiety and mood effects on emotional evaluation have been controlled by using the self-evaluation ‘Visual Analogue Scale’ (VAS) (Hayes and Paterson, 1921). The VAS consists of a horizontal line, 100 mm in length, anchored at each end by a word descriptor and the subject is required to mark on the line the point they felt best represented how they perceived their current state. The VAS score is calculated by measuring the distance from the left-hand end of the line to the point that the subject marked in millimeters.

Two VAS were used to assess the two different domains: anxiety (0 mm, no anxiety and 100 mm, the worst anxiety ever) and mood (0 mm, the worst mood and 100 mm, the best mood ever).

### Data Analyses

Non-parametric Mann- Whitney U test for independent samples was used to detect differences in accuracy row score of each test between CB patients and HS. Spearman rank-order correlation coefficient was used to correlate each test score with the VAS, the ICARS total score, the disease duration and executive function scores to exclude the possible effect of mood, motor impairment and executive function on social cognition performance. The statistical analyses were performed using Statistica software 12 (http://www.statsoft.com).

### MRI Data Acquisition Protocol

All participants underwent an MRI examination at 3T (Magnetom Allegra, Siemens, Erlangen, Germany). MRI image acquisition included the following: (1) dual-echo turbo spin echo (TSE) [repetition time (TR) = 6190 ms, echo time (TE) = 12/109 ms] and (2) T2 fluid attenuated inversion recovery (FLAIR) [TR = 8170 ms, TE = 96 ms, inversion time (TI) = 2100 ms] for conventional MRI visualization of the brain; (3) anatomical 3D Modified Driven Equilibrium Fourier Transform (MDEFT) scan [TR = 1338 ms, TE = 2.4 ms, matrix = 256 × 224 × 176, in-plane field of view (FOV) = 250 × 250 mm^2^, slice thickness = 1 mm] for structural T1-weighted imaging of the brain; (4) T2^*^ weighted echo planar imaging (EPI) sensitized to blood oxygenation level dependent imaging (BOLD) contrast [TR: 2080 ms, TE: 30 ms, 32 axial slices parallel to anterior-posterior commissure (AC-PC) line, matrix: 64 × 64, pixel size: 3 × 3 mm^2^, slice thickness: 2.5 mm, flip angle: 70°] for resting-state functional MRI (RS-fMRI).

BOLD echo planar images were collected during rest for a 7 min and 20 s period, resulting in a total of 220 volumes. During this acquisition, subjects were instructed to keep their eyes closed, not to think of anything in particular, and not to fall asleep. The absence of macroscopic extra cerebellar abnormalities was excluded by the visual inspection of the TSE and FLAIR scans of patients, acquired as part of this research study, by an expert neuroradiologist. According to the inclusion criteria, conventional MRI scans of HS were also reviewed and any pathological conditions affecting the brain was excluded.

### Image Processing

#### T1-Weighted Scans

Anatomical T1-weighted images were used to quantify the cerebellar gray matter (GM) patterns. The cerebellum was preprocessed individually using the Spatially Unbiased Infratentorial Template (SUIT) toolbox (Diedrichsen et al., 2009) implemented in Statistical Parametric Mapping version 8 [Wellcome Department of Imaging Neuroscience; SPM-8 (http://www.fil.ion.ucl.ac.uk/spm/)]. The procedure involved cropping and isolating the cerebellum from the T1 anatomical images, normalizing each cropped image into SUIT space, reslicing the probabilistic cerebellar atlas into individual subjects' space using the deformation parameters obtained by normalization, and smoothing the images using 8-mm full width at half maximum (FWHM) Gaussian kernel. Additionally, every participant's MDEFT was also segmented in SPM to estimate the total GM volume and a two sample *t*-test was performed to compared the GM total volume between groups to exclude the presence of cerebral atrophy in patients.

#### Resting-State fMRI Data

FMRI data were preprocessed using SPM8 (http://www.fil.ion.ucl.ac.uk/spm/) and in-house software implemented in MATLAB (The Mathworks Inc., Natick, Massachussetts, USA). For each subject, the first four volumes of the fMRI series were discarded to allow for T1 equilibration effects. The preprocessing steps included correcting for head motion, compensating for slice-dependent time shifts, normalizing to the EPI template in Montreal Neurologic Institute (MNI) coordinates provided with SPM8, and smoothing with a 3D Gaussian Kernel with 8 mm^3^ full-width at half maximum. For each data set, motion correction was checked to ensure that the maximum absolute shift did not exceed 2 mm and the maximum absolute rotation did not exceed 1.5°. The global temporal drift was removed using a 3rd order polynomial fit, and the signal was regressed against the realignment parameters and the signal averaged over whole brain voxels to remove other potential sources of bias. Then, all images were filtered by a phase-insensitive band-pass filter (passband 0.01–0.08 Hz) to reduce the effect of low frequency drift and high frequency physiological noise.

### Neuroimaging Data Analysis

Since 5 CB (CB3, CB13, CB23, CB32, CB34) patients did not complete the MRI protocol due to claustrophobic concerns and 2 HS were excluded from the MRI data analyses due to motion exceeding the set thresholds (2 mm translation and 1.5° rotation) during their MRI scans, only 22 CB patients (mean age/SD: 46.2/11.7; M/F: 4/18) and 25 HS (mean age/SD: 53.8/5.9; M/F: 6/19) were included in the final MRI data analyses.

#### Voxel-Based Morphometry

The individual GM maps obtained were used to perform statistical analysis and to assess differences in regional cerebellar volume between CB patients and HS by performing voxel-based morphometry (VBM) and a voxelwise two-sample *t*-test in SPM-8 to compare the GM maps. Age and sex were set as variables of no interest. The results were considered significant at *p*-values < 0.05 after familywise error (FWE) cluster-level correction.

#### Definition of Regions of Interest (ROIs) and Seed-Based fMRI Analyses

Based on the VBM results, specific cerebellar regions were identified and used as regions of interest (ROIs) in the seed-based analysis. Each cerebellar region of significantly reduced GM volume was extracted according to the SUIT atlas template of the cerebellum (Diedrichsen et al., 2009) using the FSL command line from the FMRIB software library (FSL, www.fmrib.ox.ac.uk/fsl/) and resliced into EPI standard space. The mean time course of the voxels within the affected ROI was calculated for every participant and used as a regressor in a 1st level SPM analysis, thus extracting the voxels in the whole brain showing a significant correlation with it. At the second level, a two-sample *t*-test model was used to explore differences in connectivity between CB patients and HS in the ROI. To remove the effect of confounding variables, the quantity of total brain GM volume, age and sex were entered in the analysis as covariates of no interest. The results were considered significant at *p*-values < 0.05 after FWE cluster-level correction.

## Results

### Neuropsychological Results

The performances obtained by the CB patients in the neuropsychological evaluation are reported in Table 2. The neuropsychological assessment revealed the presence of selective and very slight impairments in some patients but did not show clear evidence of general cognitive impairment. Indeed, only some patients displayed impaired performance in specific tasks, as shown in Table 2.

**Table 2:** Neuropsychological results for the CB patients.

**Neuropsychological Tests**	**Mean (sd)**	**Range**	**Cut-off**	**Number impaired**	**Not tested**
**INTELLECTUAL FUNCTIONING**					
WAIS-R	88.30 (13.11)	61–115	< 70	1 (CB35)	–
Ravens' 47	29.44 (3.20)	22–34	< 18.96	–	–
**VERBAL COMPREHENSION**					
Token test	32.50 (1.53)	29–35	< 32	1 (CB21)	1 (CB27)
**VERBAL PRODUCTION**					
Denomination of words described by the examiner	1.22 (1.51)	0–5	>2	2 (CB3, CB33)	4 (CB1, CB27, CB9, CB35)
Phrase Construction	10.64 (2.61)	3–15	< 8.72	3 (CB5, CB14, CB21)	5 (CB1, CB3, CB7, CB9, CB12)
**VERBAL MEMORY**					
Rey's 15 mots short term	44.24 (6.47)	34–61	< 28.53	–	1 (CB7)
Rey's 15 mots long term	9.92 (2.36)	5–14	< 4.69	–	1 (CB7)
Forward digit span	5.72 (0.89)	4–8	< 5	2 (CB5, CB24)	1 (CB7)
Backward digit span	4.32 (1.07)	3–7	< 3	–	1 (CB7)
**EPISODIC MEMORY**					
Short-Story recall	10.75 (3.57)	2–15	< 4.75	2 (CB4, CB33)	1 (CB7)
**VISUOSPATIAL MEMORY**					
Rey-Osterrieth figure (recall)	12.57 (6.76)	1–27	< 9.47	7 (CB3, CB4, CB14, CB21, CB29, CB30, CB32)	1 (CB7)
Forward Corsi	5.36 (1.19)	3–9	< 5	5 (CB5, CB12, CB15, CB16, CB35)	2 (CB7, CB4)
Backward Corsi	4.64 (1.11)	3–8	< 3	–	2 (CB7, CB4)
**VISUOSPATIAL ABILITY**					
Rey-Osterrieth figure (copy)	30.97 (3.60)	18–36	< 28.88	2 (CB3, CB32)	1 (CB7)
**ATTENTION**					
Multiple features targets cancellation task	0.93 (0.07)	0–1	< 0.869	2 (CB14, CB30)	1 (CB7)
Lines cancellation task	0.28 (0.74)	0–3	–	–	1 (CB7)
Trail making test:	65.13 (28.26)	29–153	≥94	1 (CB33)	3 (CB7, CB9, CB35)
A					
B	127.54 (37.14)	60–211	≥283	–	3 (CB7, CB9, CB35)
A-B	59.71 (37.01)	−8 to 178	≥187	–	3 (CB7, CB9, CB35)
**EXECUTIVE FUNCTIONS**					
Phonological fluency (FAS)	30.38 (9.04)	17–54	< 17.35	1 (CB9)	1 (CB7)
WCST:					
Total Errors	107.62 (10.48)	81–119	< 85-91	1 (CB9)	1 (CB7)
Perseverative Responses	110.58 (24.38)	81–138	< 85-91	–	1 (CB7)
Perseverative Errors	114.54 (13.71)	81–138	< 85-91	1 (CB9)	1 (CB7)

*The patients' performance to each test was considered impaired when the score was below the cut-off value, with exception of the “Denomination of words described by the examiner” and the “Trial making test” in which the performance resulted impaired when the score was higher than the cut-off value*.

### Social Cognition Profile

In the RME test, the CB patients showed an impaired performance compared to HS (MWU = 163; *Z* = −2.89; *p* = 0.004). Moreover, the patients failed in the Advanced ToM test, with a significantly lower score than HS (MWU = 255; *Z* = −2.02; *p* = 0.041). In the Faux Pas test, the patients obtained significantly lower scores than HS selectively in the “faux pas” stories (MWU = 228.5; *Z* = −2.36; *p* = 0.018), while no significant difference was observed in the “no-faux pas” stories (MWU = 347.5; *Z* = −0.23; *p* = 0.82). Normal performance was detected in the EA test total score (MWU = 310; *Z* = −0.94; *p* = 0.34). Boxplots of the row scores obtained by CB and HS in each social cognition task are reported in Supplementary Figure 1.

**Figure 1:**
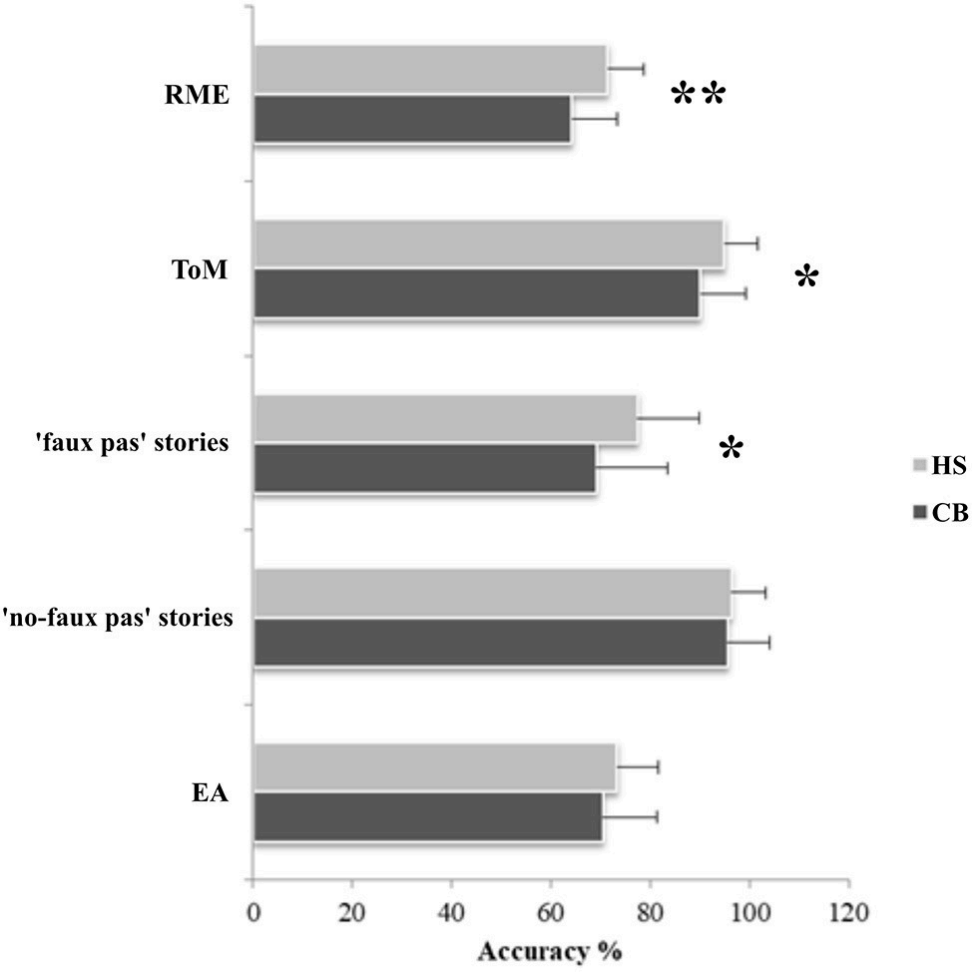
Results of the social cognition battery. Data are presented as the percentage of the total number of correct responses for the RME (max = 36), for the Advanced ToM test (max = 13), faux pas stories (max = 60) and no-faux pas stories (max = 20), and for the EA test (max = 58). Mean and standard deviation of the accuracy percentage, where 0% is totally wrong and 100% is totally correct, are reported for both patients and healthy subjects; ^*^p < 0.05; ^**^p < 0.005.

The percentage of accuracy, calculated as the percentage of the correct responses for each test, is shown in Figure 1.

No correlations were evidenced between each task score and the ICARS total score, the disease duration and executive function scores. Regarding the VAS scores, an inverse correlation was detected only between VAS-Mood and EA score. The results of Spearman correlation analyses are reported in Table 3.

**Table 3:** Correlations between each social cognition tasks score and the VAS, the ICARS total score, the disease duration and executive functions scores (WCST, FAS).

	**RME**	**ToM**	**“FP” Stories**	**“no-FP” Stories**	**EA**
VAS-Mood	*R* = −0.27	*R* = 0.11	*R* = −0.20	*R* = 0.09	*R* = −0.60
	*P* = 0.25	*P* = 0.61	*P* = 0.34	*P* = 0.68	*P* = 0.00
VAS-Anxiety	*R* = 0.02	*R* = 0.01	*R* = 0.26	*R* = −0.17	*R* = −0.22
	*P* = 0.93	*P* = 0.98	*P* = 0.22	*P* = 0.43	*P* = 0.30
ICARS Total Score	*R* = −0.07	*R* = −0.41	*R* = 0.12	*R* = −0.13	*R* = 0.11
	*P* = 0.74	*P* = 0.06	*P* = 0.54	*P* = 0.53	*P* = 0.58
Disease Duration	*R* = −0.16	*R* = −0.20	*R* = −0.02	*R* = 0.05	*R* = −0.15
	*P* = 0.50	*P* = 0.38	*P* = 0.94	*P* = 0.83	*P* = 0.50
WCST (Total Errors)	*R* = −0.08	*R* = −0.02	*R* = −0.24	*R* = 0.00	*R* = −0.03
	*P* = 0.72	*P* = 0.93	*P* = 0.25	*P* = 0.99	*P* = 0.88
WCST (Perseverative Errors)	*R* = −0.07	*R* = −0.10	*R* = −0.22	*R* = 0.01	*R* = −0.11
	*P* = 0.76	*P* = 0.64	*P* = 0.27	*P* = 0.95	*P* = 0.60
FAS	*R* = 0.02	*R* = −0.31	*R* = 0.22	*R* = 0.09	*R* = 0.05
	*P* = 0.94	*P* = 0.12	*P* = 0.29	*P* = 0.69	*P* = 0.79

*VAS, Visual Analogue Scale; ICARS, International Cooperative Ataxia Rating Scale; WCST, Wisconsin Card Sorting Test; FAS, Phonological fluency*.

### MRI Results

#### Voxel-Based Morphometry

**Figure 2:**
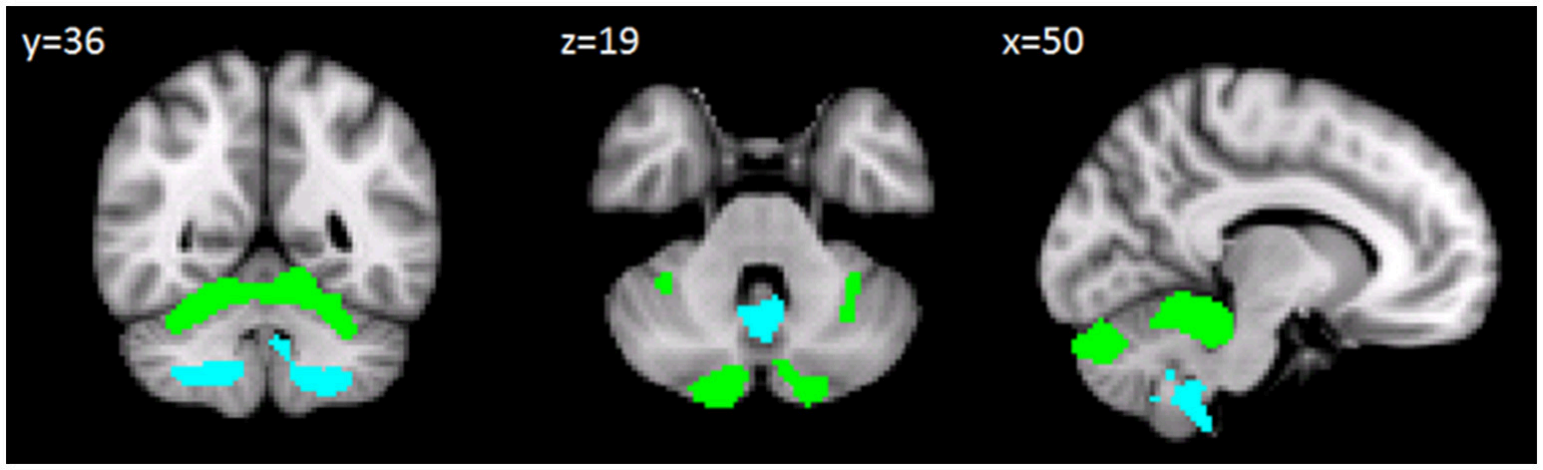
Between-group voxel-based comparison of cerebellar GM density. Cerebellar regions showing patterns of significantly reduced GM in CB compared to HS are reported and superimposed on the Spatially Unbiased Infratentorial Template (SUIT) (Diedrichsen et al., 2009) in coronal (*y* = 36), axial (*z* = 19) and sagittal (*x* = 50) slices. Clusters of significantly decreased GM in the cerebellum are shown in green (cluster size: 343334) and light blue (cluster size: 11568). The results significant at *p*-values < 0.05 after family wise error (FWE) cluster-level correction. Images are shown in radiological convention.

The between-group voxel wise comparison of the GM maps revealed a statistically significant GM loss in the cerebellar cortex of CB patients compared to HS. More specifically, a large cluster of significantly decreased GM volume (cluster size: 34334; FWE *p* = 0.05) was found. Peak voxels were centered in the left and right lobules I-IV of the anterior cerebellum and right lobule VI with extension in the left side and vermis portion and in the left and right crus I-II. A second large cluster of significantly decreased GM volume (cluster size: 11568; FWE *p* = 0.05) was also found. Peak voxels were centered in the bilateral hemispheric lobule VIIIa as well as vermis VIIIa with extension in vermis IX (Figure 2). Detailed results with peak voxel coordinates of voxel wise analyses are reported in Table 4.

**Table 4:** Statistics of voxel wise comparisons of cerebellar GM density (CB < HS).

**Cluster Size** ** (NoV)**	**Coordinates**			**Cluster Peak *Z*-score**	**Brain region**
	***x***	***y***	***z***		
34334	−10	−38	−19	5.23	L-Hem I-IV
	13	−38	−22	5.12	R-Hem I-IV
	22	−63	−25	4.66	R-Hem VI
11568	−23	−57	−50	4.65	L-Hem VIIIa
	0	−59	−34	4.51	R-Hem VIIIa
	23	−58	−49	4.50	Vermal-VIIIa

No significant differences were found between total GM volumes of CB patients (mean = 645.91 mm^3^; *SD* = 68.05) and HS (mean = 656 mm^3^; *SD* = 49.93) as assessed by the *t*-test analysis (*t*-value 0.8567; *p* = 0.39).

#### Seed-Based fMRI Analysis

**Figure 3:**
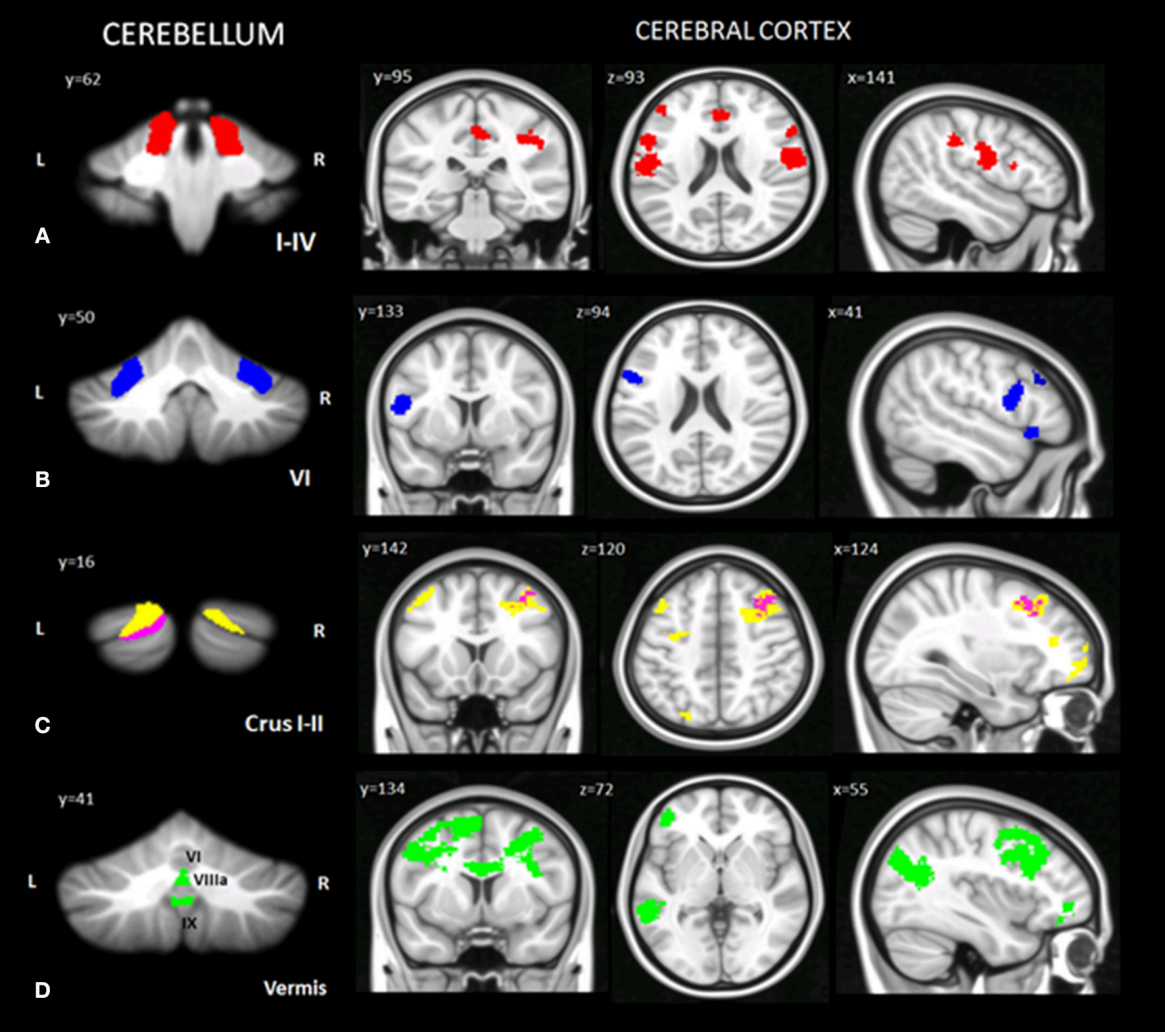
Cerebello-cerebral functional connectivity. Cerebellar regions of interest and corresponding cluster of decreased functional connectivity in the cerebral cortex. **(A)** Anterior cerebellar ROIs (I-IV, in red); **(B)** intermediate cerebellar ROIs (VI, in blue); **(C)** posterior cerebellar ROIs (crus I, in yellow; crus II, in magenta); **(D)** vermal cerebellar ROIs (VI, VIIIa, IX, in green). Cluster of significantly decreased functional connectivity in the cerebral cortex of patients, shown in coronal (y), axial (z), and sagittal (x) slices. Cluster-level FWE correction (*p* < 0.05). Detailed statistics and coordinates of the peak voxels showing statistical significance in the cluster are reported in Table 5. *R* = right, *L* = left.

Taking into account the VBM results, specific cerebellar regions of reduced GM were chosen as ROIs for the seed-based analysis (see section Materials and Methods). A total of 13 different voxel wise analyses were performed. When comparing CB patients and HS, selectively in the CB patients, distinct patterns of significantly decreased FC were found between cerebellar ROIs and the cerebral cortex (Figure 3; Table 5).

**Table 5:** Statistics of cerebellar ROI functional connectivity results (CB < HS).

		**Cluster Size** ** (NoV)**	**Coordinates**			**Cluster Peak *Z*-score**	**Brain region**	**Brodmann areas**
			***x***	***y***	***z***			
Anterior	Left I-IV	1024	−48	−20	28	4.97	L- Postcentral Gyrus	3
			−54	12	8	4.58	L- Inferior Frontal Gyrus	44
			−52	10	18	4.18		
		493	−38	50	0	4.66	L- Middle Frontal Gyrus	46
			−40	46	10	4.41		
			−36	42	2	4.23	L- Orbitofrontal cortex	47
		335	50	−30	36	4.39	R- Postcentral Gyrus	2
			38	−32	40	3.98	R- Supramarginal Gyrus	40
			40	−40	46	3.95		40
		240	58	24	22	4.35	R- Inferior Frontal Gyrus	45
			60	2	10	4.08	R- Rolandic Operculus	–
			50	−6	14	3.23		
		302	−6	36	14	4.03	L- Anterior Cingulate	24
			6	36	8	3.53	R- Anterior Cingulate	25
			6	36	24	3.52		32
	Right I-IV	1189	−42	44	10	4.73	L- Inferior Frontal Gyrus	45
			−38	42	0	4.57	L- Orbitofrontal Cortex	47
			−38	50	2	4.33	L- Middle Frontal Gyrus	46
		906	52	−4	20	4.61	R- Rolandic Operculum	–
			58	20	16	4.45	R- Inferior Frontal Gyrus	44
			66	−12	24	3.99	R- Postcentral Gyrus	43
		1411	−54	14	8	4.38	L- Inferior Frontal Gyrus	44
			−48	−8	24	4.28	L- Precentral Gyrus	4
			−46	−18	28	4.26	L- Postcentral gyrus	3
		182	6	−28	44	3.90	R- Posterior Cingulate	23
			14	−22	46	3.62		
			18	−28	42	3.35		
Intermediate	Left VI	192	−44	26	28	4.24	L- Middle Frontal Gyrus	46
			−48	26	36	4.17		
			−42	32	38	3.47		
	Right VI	447	−50	6	18	4.28	L- Precentral gyrus	6
			−52	20	−8	3.70	L- Temporal Pole	38
			−54	12	24	3.60	L- Inferior Frontal Gyrus	44
Posterior	Left Crus I	295	−34	6	56	4.85	L- Middle Frontal Gyrus	8
			−44	20	46	4.04	L- Middle Frontal Gyrus	9
		1006	36	26	42	4.80	R- Middle Frontal Gyrus	9
			28	22	40	4.77		
			28	8	42	4.40		
		406	26	54	0	4.71	R- Orbitofrontal Cortex	11
			30	50	−10	3.56		
			28	28	12	4.21	R- Superior Frontal Cortex	10
		246	48	36	16	3.71	R- Inferior Frontal Gyrus	45
			30	34	16	3.68		
			36	40	16	3.67		
	L-Crus II	366	30	22	44	4.58	R- Middle Frontal Gyrus	8/9
			46	24	40	4.18		
			36	18	48	3.96		
	R-Crus I	239	−46	28	14	4.50	L- Inferior Frontal Gyrus	45
		210	−30	−14	56	3.96	L- Precentral Gyrus	6
			−34	−8	42	3.89		
			−34	4	62	3.77		
Vermis	VIIIA	7964	−44	20	28	4.43	L - Inferior Frontal Gyrus	44
			36	14	30	3.77	R - Inferior Frontal Gyrus	44
			−16	10	60	3.60	L - Superior Frontal Gyrus	6
		2783	−40	58	16	3.79	L- Frontal Pole	46
			−34	−56	32	3.77	L - Angular Gyrus	19
			−58	−42	−6	3.66	L- Middle Temporal Cortex (STS)	21/22
	VI	228	−42	48	−4	4.36	L- Frontal Pole	47
			−40	40	−16	3.89	L- Frontal Pole	47
			−30	50	−10	3.68	L- Frontal Pole	11/47
		202	26	8	44	4.27	R- Middle Frontal Gyrus	6/8
			34	8	52	4.02	R- Middle Frontal Gyrus	9
			16	2	40	3.55	R- Anterior Cingulate	24
		387	−12	8	50	4.11	L- Supplementaly Motor Area	6
			−16	12	64	4.01		
			−10	8	58	3.79		
	IX	635	−30	20	56	3.78	L- Middle Frontal Gyrus	8
			−40	12	54	3.77	L- Middle Frontal Gyrus	9
			−16	16	46	3.56	L- Anterior Cingulate	32

*MNI coordinates (x, y, z) in the Montreal Neurological Institute space and peak Z score of the peak voxels showing the greatest statistical differences in a cluster are reported. Only regions that survived after correction for multiple comparisons (FWE corrected p < 0.05) were considered. NoV = number of voxels; L: left; R: right*.

In the anterior cerebellum, lobules I-IV showed decreased FC with cortical regions related to motor and somatosensory control, such as the precentral gyrus (BA 4, 6), postcentral gyrus (BA 3, 43), rolandic operculum, and inferior frontal gyrus (BA 44, 45). Moreover, decreased FC was also evidenced with cerebral areas involved in mentalizing processes, such as the supramarginal gyrus, anterior cingulate cortex (BA 24, 32), posterior cingulate cortex (BA 23) (right lobule I-IV), left orbitofrontal cortex (BA 47) and middle frontal gyrus (dorsolateral prefrontal cortex - dlPFC - BA 46) (Figure 3A).

In the intermediate cerebellum, lobule VI showed a decreased FC with the middle frontal gyrus (dlPFC - BA 46), left premotor cortex (BA 6), inferior frontal gyrus (BA 44) and temporal pole area (BA 38) (Figure 3B).

In the posterior cerebellum, crus I-II showed decreased FC with cortical regions implicated in more complex and abstract aspects of social cognition. In particular, decreased FC was found between the left crus I-II and the middle frontal gyrus, the dorsomedial prefrontal cortex (dmPFC) (BA 8, 9), the superior frontal gyrus (BA 10) and the orbitofrontal cortex (BA 11). Reduced FC was also present between the right crus I and left inferior frontal gyrus (BA 45) and precentral gyrus (in the supplementary motor area - SMA - BA 6) (Figure 3C).

No significant functional alterations were found between the right crus II, the right and left lobule VIIIa and the cerebral cortex.

Finally, decreased FC was evidenced between specific portions of the vermis and cerebral areas involved in emotional processing or belonging to mirroring and mentalizing networks, such as the middle frontal gyrus (dmPFC - BA 9), anterior cingulate cortex (BA 32, 24), premotor cortex and supplementary motor areas (BA 6, 8), orbitofrontal cortex (BA 11, 47), inferior frontal gyrus (BA 44), middle frontal gyrus (dlPFC - BA 46), angular gyrus, and superior temporal sulcus (STS) (BA 21/22) (Figure 3D).

A detailed report of the seed-based analyses with MNI coordinates, and peak Z scores is summarized in Table 5, where the numbers of voxels in each cluster express the extension and magnitude of significant FC modifications and the peak z-scores express the highest significance in a voxel.

## Discussion

In recent decades, the cerebellum has been acknowledged as a central area in the context of adaptive control and predictive coding, including the prediction and organization of sensorimotor and cognitive behavior (Ito, 2008; Molinari et al., 2009; D'Angelo and Casali, 2013; Sokolov et al., 2017).

In the present study, we used well-known social cognitive tasks focusing on different anticipation/prediction requirements to clarify the possible role of the cerebellum as a predictor in social interactions. As previously stated, in social interactions, at least two distinct processes are fundamental: lower-level processes of immediate perception that include an immediate affective response (i.e., the visceral feelings perceived when we look at another fearful, smiling or crying person) and a more reflective and conscious representation based on the role and perspective taking (i.e., the capacity to suppose and understand why a person is scared, happy, or sad) to make predictions about imminent or future social behavior (Coricelli, 2005; Shamay-Tsoory et al., 2009).

Interestingly, we found that our cohort of patients presented with alterations both in the immediate and automatic perception of emotion and mental state and in the more complex conceptual level of ToM process. Specifically, the CB patients showed an impaired performance in the RME test, which involves the automatic attribution of relevant mental states regardless of the context. The RME test requires the subjects to “tune in” to the mental state of the actor's eye-region expression at an unconscious, rapid, and automatic level (Baron-Cohen et al., 2001). In this case, automaticity and categorization are crucial to determine the meaning of expression (Knutson et al., 2007) and to infer the other's mental state (Hoche et al., 2016).

Looking at the more complex level of the mentalizing process, the CB patients showed impairments in the Advanced ToM task and in the social “faux pas” stories (Stone et al., 1998; Blair and Cipolotti, 2000). In these conditions, the sequential events are unexpected and not univocal, requiring a constant comparison between the event and the social expectation and a high level of prediction. For example, the detection of a “faux pas” (i.e., when someone says something they should not and not realizing they should not say it) requires not only the cognitive understanding that a person has said something inappropriate with respect to the expected behavioral patterns but also the prediction of the consequences of the actor's behavior. The subject is required to predict the actor's behavior based on previous experiences to recognize the upcoming error.

When the patterns of the stories required a minor level of prediction and of error monitoring, such as in the “no-faux pas” stories and in the Emotion Attribution test, the CB patients showed good performance. Indeed, in these conditions, the social situation was univocal and well described in the story text.

Our results are in line with earlier reports of patients affected by cerebellar pathology that revealed specific deficits in the RME test (Hoche et al., 2016) and in social emotion identification from faces (D'Agata et al., 2011; Adamaszek et al., 2014). Moreover, an impairment in the Advanced ToM task was found in patients with superficial siderosis (a pathological condition predominantly involving the cerebellum), despite normal performance on the Emotion Attribution test and social judgment tasks (Van Harskamp et al., 2005).

In our cohort of CB patients, the alterations in specific aspects of ToM are not explained by a generalized intellectual and/or a verbal comprehension impairment. Indeed, the neuropsychological assessment revealed the presence of selective and very slight impairments in some patients but did not show clear evidence of general cognitive impairment. This result is consistent with findings that patients who are affected by cerebellar damage do not present with intellectual deterioration (Tedesco et al., 2011). It is worth noting that in cerebellar patients' cohorts, mostly standard norms of testing do not detect cognitive impairments characterizing the “cerebellar cognitive affective syndrome” (CCAS) (Schmahmann and Sherman, 1998), and very often they can be detected only when the patients are compared to matched healthy controls. In this respect, a scale was recently published to diagnose the CCAS (Hoche et al., 2018), but we unfortunately collected our data before its publication. Moreover, our sample had no difficulties in understanding other story-type stimuli, such as “no-faux pas” stories or emotion attribution tasks. Additionally, it is also unlikely that a generalized executive impairment accounts for our patients' mentalizing deficit, considering the absence of correlation between the ToM scores and the executive test scores in our cohort. This claim is in line with the increasing literature suggesting that executive functioning and ToM abilities are dissociable (Blair and Cipolotti, 2000; Fine et al., 2001; Bird et al., 2004). Moreover, the correlation analysis excluded any relationship between mood and motor impairment on task performance. It has to be noted that the correlation between the EA score and the VAS-Mood score was inverse; thus, it does not explain CB performance in EA test.

Altogether, these findings indicated that in the presence of cerebellar damage, the performances subtending the social interaction become less accurate mainly when the stimuli require automatic processing or a high level of prediction.

Particularly, regarding the automatic processing and the well-known cerebellar role in implicit elaboration (Molinari et al., 2002; Schmahmann, 2018), the cerebellum may act at the implicit level by matching the external information (i.e., expression of the eyes) with the internal model of eye-region expression linked to previous emotional experiences, contributing to guarantee an immediate judgment about the mental state of others. When cerebellar damage is present, the required fast and continuous exchange of information between the external stimuli and the internal model might be affected, thus interfering with the automatic processes.

At a more complex level, to obtain a sense of another individual's state of mind, we need to predict the social consequences of how we act or what we say across various contexts, and we have to understand what caused a specific behavior and how it may impact the social situation (Mahon and Caramazza, 2008; Koster-Hale and Saxe, 2013). In this context, the capacity to recognize deviations/errors in the outcome of a social interaction and to use this information to regulate and adjust future social expectations becomes useful for adaptive social behavior.

To study error processing in the ToM domain, Berthoz et al. (2002) used a contrast between scenarios in which a social prediction was confirmed or violated. They found that violations of social norms elicited higher activation not only in a frontotemporal network associated with social cognition but also in the cerebellum. According to the forward model theory (Ito, 2008), signals from the cerebellum might continuously check whether an anticipated event based on social information fits with current behavior, contributing to the more complex and abstract forms of prediction and guaranteeing fluid control in social interactions. In the presence of cerebellar dysfunction, the error signal is missed, and the performance becomes less accurate when the level of predictive load is high.

The present data are in line with the idea that the cerebellum plays a role both in implicit processes and in prediction mechanisms (Doyon et al., 2003) and reinforce the idea that the cerebellum can be conceptualized as a unique predictive structure in different domains and that its functional role in social cognition is similar to that for sensorimotor control (Wolpert and Kawato, 1998; Ito, 2008; Sokolov et al., 2017).

In the social domain, the processing mechanisms are supported by the bidirectional anatomical connections of the cerebellum with limbic areas and specific portions of the frontal and temporo-parietal lobes that are involved in emotional regulation and in the perception of socially salient material (Schmahmann, 1991; Schmahmann and Pandya, 1997; Middleton and Strick, 2001; Kelly and Strick, 2003). Coherently, in the present study, CB patients showed structural and functional alterations within cerebello-cortical networks that are involved in different aspects of social interactions. Specifically, in our patients, cerebellar atrophy, in terms of GM reduction, was localized in specific portions of the vermis (VI, IX, VIIIa) and in lobule VI.

fMRI studies demonstrated that these cerebellar areas are activated during classic mirror tasks (Van Overwalle et al., 2014) and belong to the Salience Network (SN) as well as to the Default Mode Network (DMN) (Habas et al., 2009; Buckner et al., 2011). Moreover, the posterior vermis is recruited during emotional processing (Baumann and Mattingley, 2012), and indeed, it is considered the “limbic cerebellum” (Schmahmann, 2007; Stoodley and Schmahmann, 2010).

In our cerebellar patients, these regions of reduced GM also showed decreased FC with cerebral areas involved in mirroring, emotional and mentalizing processing, such as the middle frontal gyrus, precentral gyrus, premotor cortex, orbitofrontal cortex, anterior cingulate, inferior frontal gyrus, angular gyrus, STS and temporal pole (Abu-Akel and Shamay-Tsoory, 2011). Indeed, it has been evidenced that higher-order cortices in the temporal pole, orbitofrontal cortex and inferior frontal gyrus subserve the processing of emotions from facial expression and in emotional contagion (Adolphs, 2004; Chakrabarti et al., 2006; Dapretto et al., 2006; Gazzola et al., 2006; Jabbi et al., 2007; Ross and Monnot, 2008; Shamay-Tsoory, 2011). The superior temporal sulcus and premotor cortex belong to the mirror network (Bickart et al., 2014). The medial prefrontal cortex, medial temporal lobe and angular gyrus belong to the DMN, which is thought to be involved in mental simulation for planning, self-evaluation, and social interaction (Habas et al., 2009).

We also found a pattern of GM reduction in the anterior cerebellum (bilateral lobule I-IV). These cerebellar regions are more involved in somatosensory and motor control aspects (Schmahmann, 2010; Stoodley and Schmahmann, 2010). As expected, these lobules showed reduced FC with the precentral gyrus, postcentral gyrus, and rolandic operculum. However, we also observed a pattern of decreased FC between the anterior cerebellum and cerebral areas involved in the mirror network and mentalizing processes, such as the inferior frontal gyrus, supramarginal gyrus (Reed and Caselli, 1994; Carlson, 2012), anterior and posterior cingulate cortex, orbitofrontal cortex and middle frontal gyrus (in the dlPFC).

Finally, the posterior cerebellum showed a specific pattern of GM reduction mainly localized in the bilateral crus I/II. The posterior lateral cerebellum has been described as involved in more reflective, cognitive components of the mentalizing tasks (Sokolov et al., 2017). Interestingly, our CB patients showed a pattern of decreased FC between these posterior regions of the cerebellum and areas of the cerebral cortex involved in high-order social behavior and executive control, such as the dmPFC and the superior frontal gyrus and orbitofrontal cortex (Habas et al., 2009; Abu-Akel and Shamay-Tsoory, 2011; Shamay-Tsoory, 2011; Bickart et al., 2014).

Overall, in our patients, the structural alterations in the specific lobule of the cerebellum interfered with the modulatory function that the cerebellum exerts on the cortical projection areas, thus accounting for altered functional connectivity in the cerebellar-cortical networks involved in different aspects of social cognition and, in particular, in the mentalizing process. Indeed, the disrupted cerebellar modulatory function resulted in impaired ToM outcomes, particularly when the stimuli processing requires a high level of prediction.

In light of the present observations, the cerebellum could be conceptualized as a part of the social brain by virtue of the role that it plays in supporting other more classically social regions (Jack and Morris, 2014). Particularly, the cerebellar-cerebral networks could have a role in the predictive aspects of social behavior by guaranteeing the continuous communication between cerebellar modules and projection cerebral areas. Therefore, important theoretical breakthroughs can be made by studying cerebellar function in social behavior from the prediction perspective.

## Limitations

The present study is correlative at descriptive level and does not bring evidence of a direct link between the observed atrophies in the cerebellum and the reported performances in social and cognitive tasks. This is a limitation due to the heterogeneity of the study population and needs to be addressed in patients affected by homogeneous cerebellar pathologies.

Another important issue that needs to be discussed is that, even if in the present study the macroscopic damage of cerebral cortex was excluded by the visual inspection of the clinical MRI scans by an expert neuroradiologist and there was not a significant difference in the total GM volume between CB patients and HS, the possibility of local and microscopic GM loss, as reported in previous studies (Brenneis et al., 2003; Della Nave et al., 2008; Selvadurai et al., 2016) cannot be ruled out.

However, it has to be noted that the aim of the present study was to investigate the cerebello-cerebral functional connectivity that it is particularly suitable for the study of the cerebellum, in which the function of each sub-region is defined by its connections with specific brain areas (Schmahmann and Pandya, 1997; Middleton and Strick, 2001).

## Conclusion

In conclusion, in the presence of cerebellar damage, patients fail in both automatic lower-level and conceptual/abstract components of social cognition, and the idea can be advanced that the cerebellar modulatory function on the cortical projection areas subtends these processes. These findings can be explained considering different aspects of the prediction mechanisms needed for the social interactions (Brown and Brüne, 2012) and taking into account the connections that the cerebellum has with limbic areas and specific portions of the frontal and temporo-parietal lobes involved in metalizing processes (Schmahmann and Pandya, 1997; Middleton and Strick, 2001). According to the “sequence detection theory” (Braitenberg et al., 1997; Leggio et al., 2011; Leggio and Molinari, 2015), during social interactions in which event sequences play a role, the cerebellum allows the prediction of the other person's behaviors in an intuitive way to optimize the social behavior.

## Author Contributions

SC and MAL contributed to conception and design of the study. SC, MIL, and LS, contributed to the data acquisition and data analysis. GO acquired the MRI protocol, processed and analyzed the MRI data. MB supervised MRI data processing. SC wrote the first draft of the manuscript. MAL supervised development of the work. All co-authors contributed to final editing and critical revision of the original manuscript.

### Conflict of Interest Statement

The authors declare that the research was conducted in the absence of any commercial or financial relationships that could be construed as a potential conflict of interest.

## Supplementary Material

Supplementary Figure 1 Boxplots of the row scores obtained by the cerebellar patients and healthy subject in each social cognition task. CB, cerebellar patients; HS, healthy subjects; SE, standard error; SD, standard deviation.

## Abbreviations

AC-PC: anterior-posterior commissure
BA: Brodmann area
BADA: Batteria per l'Analisi dei Deficit Afasici (Battery for the analysis of the aphasic deficits)
BOLD: blood oxygenation level dependent imaging
CB: cerebellar patients
dlPFC: dorsolateral prefrontal cortex
dmPFC: dorsomedial prefrontal cortex
DMN: Default Mode Network
EA: Emotion Attribution test
EPI: echo planar imaging
FC: functional connectivity
FLAIR: fluid attenuated inversion recovery
FOV: field of view
FP: Faux Pas test
FWE: familywise error
FWHM: full width at half maximum
GM: gray matter
HS: healthy subjects
ICARS: International Cooperative Ataxia Rating Scale
MDEFT: Modified Driven Equilibrium Fourier Transform
MNI: Montreal Neurologic Institute
RME: Reading the Mind in the Eyes test
ROIs: regions of interest
RS-fMRI: resting-state functional magnetic resonance imaging
SMA: supplementary motor area
SN: Salience Network
SPM-8: Statistical Parametric Mapping version 8
STS: superior temporal sulcus
SUIT: Spatially Unbiased Infratentorial Template
TE: echo time
TI: inversion time
TR: repetition time
ToM: theory of mind
TSE: turbo spin echo
VAS: Visual Analogue Scale
VBM: voxel-based morphometry.
