# Supplementary material for: The Cerebellar Predictions for Social Interactions: Theory of Mind Abilities in Patients With Degenerative Cerebellar Atrophy
Source: Front Cell Neurosci. 2019 Jan 8;12:510. doi: 10.3389/fncel.2018.00510 (PMC6332472; doi:10.3389/fncel.2018.00510)
Supplement: Supplementary file 1 [file Data_Sheet_1.docx]

**Appendix**

**Theory of Mind Task**

Example of story: ‘Today James is going to Clare’s house for tea. He is looking forward to seeing Clare’s dog, which she talks about all the time. James likes dogs very much. When James arrives at Clare’s house, Clare runs to open the door and her dog jumps up to greet James. Clare’s dog is huge, it is almost as big as James! When James sees Clare’s huge dog he says, ‘Clare you haven’t got a dog at all. You have got an elephant.’

Comprehension question: Is what James says true?

Theory of mind question: Why does James say this?

Example of a physical interpretation response: “Because the dog is as big as an elephant’.

Example of a mental state interpretation response: ‘He is teasing her.’

**Faux Pas Test**

Example of ‘Faux Pas story’: Helen's husband was throwing a surprise party for her birthday. He invited Sarah, a friend of Helen's, and said, "Don't tell anyone, especially Helen." The day before the party, Helen was over at Sarah's and Sarah spilled some coffee on a new dress that was hanging over her chair. "Oh!" said Sarah, "I was going to wear this to your party!" "What party?" said Helen. "Come on," said Sarah, "Let's go see if we can get the stain out."

1. Did anyone say something they shouldn't have said or something awkward?

If yes, ask:

2. Who said something they shouldn't have said or something awkward?

3. Why shouldn't he/she have said it or why was it awkward?

4. Why do you think he/she said it?

5. Did Sarah remember that the party was a surprise party?

6. How do you think Helen felt?

Control question:

1. In the story, who was the surprise party for?

2. What got spilled on the dress?

Example of ‘no Faux Pas story’: Vicky was at a party at her friend Oliver’s house. She was talking to Oliver when another woman came up to them. She was one of Oliver’s neighbours. The woman said, "Hello," then turned to Vicky and said, " I don't think we've met. I’m Maria, what's your name?" "I’m Vicky." "Would anyone like something to drink?" Oliver asked.

1. Did anyone say something they shouldn't have said or something awkward?

If yes, ask:

2. Who said something they shouldn't have said or something awkward?

3. Why shouldn't he/she have said it or why was it awkward?

4. Why do you think he/she said it?

5. Did Vicky and Maria know each other?

6. How do you think Vicky felt?

Control questions:
1. In the story, where was Vicky?

2. Who was hosting the party?

**Emotion Attribution test**

Example of a story: A man walks up to Larry and calls him an idiot.

Question: How will Larry feel in this situation?
