# Supplementary figures and images for: The Cerebellar Predictions for Social Interactions: Theory of Mind Abilities in Patients With Degenerative Cerebellar Atrophy
Source: Front Cell Neurosci. 2019 Jan 8;12:510. doi: 10.3389/fncel.2018.00510 (PMC6332472; doi:10.3389/fncel.2018.00510)

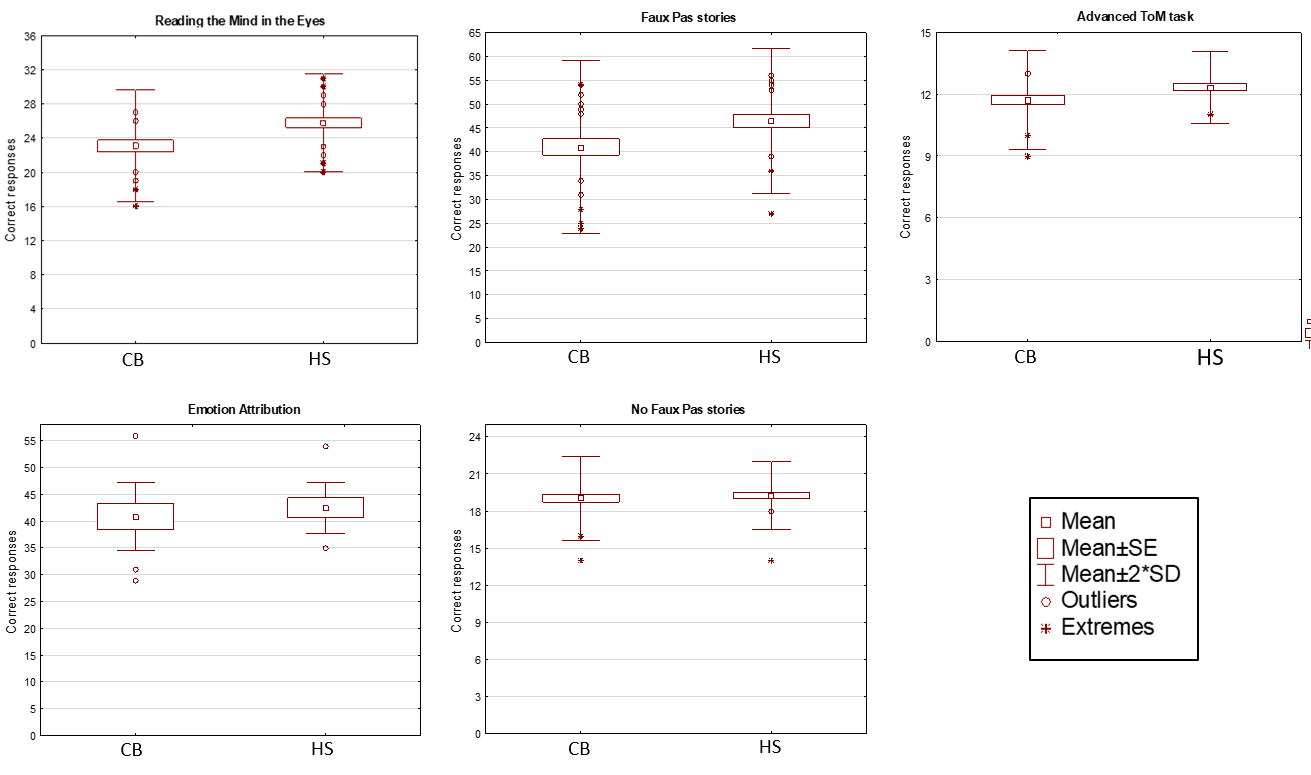

Supplement: Supplementary Figure 1 — Boxplots of the row scores obtained by the cerebellar patients and healthy subject in each social cognition task. CB, cerebellar patients; HS, healthy subjects; SE, standard error; SD, standard deviation. [file Image_1.TIF]
